# Review of *“Trypanosomes and Trypanosomiasis”* by Stefan Magez and Magdalena Radwanska (Editors)

**DOI:** 10.1186/1756-3305-6-365

**Published:** 2013-12-26

**Authors:** Nick Golding

**Affiliations:** 1 Spatial Ecology and Epidemiology Group, Department of Zoology, University of Oxford, South Parks Road, Oxford OX1 3PS, UK

**Keywords:** Trypanosoma, Trypanosomiasis, African sleeping sickness

## Abstract

**Book details:**

Magez, S and Radwanska, M (Eds.): *Trypanosomes and Trypanosomiasis* Springer-Verlag; 2014. 294 pages. ISBN 978-3-7091-1556-5

## Main text

Trypanosomiasis is responsible for significant mortality, morbidity and economic losses throughout sub-Saharan Africa and Latin America. The biology of the trypanosome parasites which cause the disease is also fascinating. Trypanosomes have evolved sophisticated stragies to evade the mammalian immune system and developed complex interactions with their insect vectors. Recent years have seen increasing research into the trypanosomiases, with progress in the control of human African trypanosomiasis paralleled by advances in basic research into parasite biology which have identified potential mechanisms for interrupting transmission. Given the pace of change of research in this area an update to the existing texts on these twin subjects is welcome.

Given its title, *Trypanosomes and Trypanosomiasis* would appear to be the book to provide such an update. Unfortunately this title is misleading and the book in fact focusses largely on the biology (primarily from a laboratory perspective) of trypanosome species of sub-Saharan Africa. The book’s most alarming omission is of any reference to American trypanosomiasis (Chagas disease, caused by the trypanosome species *Trypanosoma cruzi*). Chagas disease is estimated to have been responsible for over 10,000 deaths in 2010 - slightly more than were caused by African trypanosomiasis [1]. American trypanosomiasis also has distinct biology, ecology and epidemiology from the African trypanosomiases and it would have been interesting to have seen biological and epidemiological perspectives on both sets of diseases.

Geographical bias aside, the book focusses very heavily on the biology of a single parasite species: *T. brucei*, with the first seven chapters providing detailed accounts covering cell biology, antigenic variation and immunology. These chapters are excellently written; each is packed with detail and punctuated with clear and informative figures. Chapter 2 is a particular highlight, giving a comprehensive review of *T. brucei*’s interactions with the tsetse fly: evading the immune system, modifying biting behaviour to its benefit and interacting with other gut microflora.

By comparison with this wealth of biological information, it is unfortunate that only four chapters are given over to the study of trypanosomiasis. Whilst chapters 8 and 9 do provide a thorough overview of the historical and current state of diagnostics and treatments for human (and to a lesser extent animal) trypanosomiasis in Africa, they lack the informative visuals of the earlier chapters. The remaining two chapters give a slightly dry run-down of the animal trypanosomiases present in the Old World and Latin America and seem to have been included as an afterthought. A great deal of current epidemiological research into African trypanosomiasis is omitted. There is no mention of the ecology of the African trypanosomiases and their tsetse fly vectors, nor of the surveillance and control strategies which have led to a fourfold reduction in human disease burden over the last 20 years.

This is clearly a book compiled by and for laboratory-based biologists, rather than an overview of the entire research area. Set within that scope it is a useful reference text of the current state of knowledge on the biology of *T. brucei* and can be recommended to those with an interest in this area. When it comes to trypanosomiasis or other trypanosome species however this book is no replacement for any of the existing comprehensive (though already fairly dated) tomes on the subject.

## Competing interests

The author declares that they have no competing interests.
